# A Recent Treatment for Irreducible Retroflexion of the Uterus

**Published:** 1877-03-20

**Authors:** A. H. Goelet


THE
### SOUTHERN MEDICAL RECORD.

VOL. VII. ATLANTA, GA., MARCH 20, 1877. No. 3.

THOMAS S. POWELL, M. D., EDITOR.
W. T. GOLDSMITH, M. D., Assistant Editor.
R. C. WORD, M. D., Assistant Editor and Business Manager.

SUBSCRIPTION: TWO DOLLARS PER ANNUM, IN ADVANCE.

☞ All communications, and letters on business connected with THE RECORD for 1877 must be addressed to the Business anager.

### Original and Selected Articles.

### A RECENT TREATMENT FOR IRREDUCIBLE RETROFLEXION OF THE UTERUS.

BY A. H. GOELET, M. D,

By Irreducible Retroflexion is meant a retroflexed Uterus, either congenital or accidental, which has been bound down in this abnormal position by false membranous bands, the result of subsequent pelvic inflammation. Most authors on gynæcology, say that we can only resort to palliative means in these cases. Dr. Thomas in his excellent work on diseases of Women, advises that the uterus be left in its abnormal position unless the symptoms are such as to render reposition urgently necessary, in which case he thinks it should be forcibly replaced. But this latter procedure subjects the patient to the dangers of peritonitis. Prof. Carl Schrœder, who writes the volume on diseases of the Female Sexual Organs for Ziemssen's Cyclopedia of Medicine, says in treating of this subject: "If the conditions of the displacement are such as to preclude any attempt at replacement, we are restricted to merely palliative treatment, which in most cases avails to render the patient tolerably comfortable." All other writers whose works I have consulted hold the same opinion.

Dr. Emmet makes a step in advance and treats these cases by introducing the Uterine Repositor or the sound twice or three times a week, and gently forces the uterus upward and forward, thus stretching these membranous bands a little more every time. This, in the end. results in a cure, but has the disadvantage of being long and tedious to both physician and patient, and particularly objectionable because it necessitates *too much treatment*. There will be found few patients who will follow this out to the end.

Now what I have found equally successful, and less objectionable, is this: Without attempting replacement of the organ, introduce an ordinary Hodge's closed lever pessary, which has been perfectly fitted to the particular case, so that the patient may wear it with perfect ease and comfort. This will produce a constant stretching of these false attachments by steady pressure on the fundus upward, and will, if worn sufficiently long, accomplish the same purpose that Dr. Emmet does with the repositor; thus avoiding the objection of a too frequent use of that instrument, of which the patient will soon weary.

To verify the success of this procedure, I will quote one case from my private practice: Miss Anna D. aged 19 years, unmarried, dressmaker, consulted me in December, 1875. She dated all her ailments about two years back, when she had a severe attack of illness, and her physician told her she had "inflammation of the womb." Since this she had always suffered from dysmenorrhœ, lucorrhœa, intense backache, headache, and constipation; and when her bowels were moved she experienced most excrutiating pain. Upon examination I found the uterus retroflexed and its reduction impossible. Dr. Geo. T. Harrison of this city saw her in consultation, and agreed with me that it was an irreducible flexion. I did not attempt further replacement, but fitted and introduced a Hodge's closed lever pessary, which she wore without any inconvenience. I gave a laxative pill to regulate the bowels, and I saw her once or twice a month for about three months. After this she passed from under my observation and did not return again 'till October 9th, 1876, when I removed the pessary; and on introducing the sound the uterus was found in its normal position. All her disagreeable symptoms had disappeared.

